# Assessment of vestibulocortical interactions during standing in healthy subjects

**DOI:** 10.1371/journal.pone.0233843

**Published:** 2020-06-04

**Authors:** Jean-François Nepveu, Youstina Mikhail, Charlotte H. Pion, Jean-Pierre Gossard, Dorothy Barthélemy

**Affiliations:** 1 Centre for Interdisciplinary Research in Rehabilitation of Greater Montreal, CRIR, Montreal, Canada; 2 Department of Neuroscience, Université de Montréal, Montreal, Canada; 3 School of Rehabilitation, Université de Montréal, Montreal, Canada; BG-Universitatsklinikum Bergmannsheil, Ruhr-Universitat Bochum, GERMANY

## Abstract

The vestibular system is essential to produce adequate postural responses enabling voluntary movement. However, how the vestibular system influences corticospinal output during postural tasks is still unknown. Here, we examined the modulation exerted by the vestibular system on corticospinal output during standing. Healthy subjects (n = 25) maintained quiet standing, head facing forward with eyes closed. Galvanic vestibular stimulation (GVS) was applied bipolarly and binaurally at different delays prior to transcranial magnetic stimulation (TMS) which triggered motor evoked potentials (MEPs). With the cathode right/anode left configuration, MEPs in right Soleus (SOL) muscle were significantly suppressed when GVS was applied at ISI = 40 and 130ms before TMS. With the anode right/cathode left configuration, no significant changes were observed. Changes in the MEP amplitude were then compared to changes in the ongoing EMG when GVS was applied alone. Only the decrease in MEP amplitude at ISI = 40ms occurred without change in the ongoing EMG, suggesting that modulation occurred at a premotoneuronal level. We further investigated whether vestibular modulation could occur at the motor cortex level by assessing changes in the direct corticospinal pathways using the short-latency facilitation of the SOL Hoffmann reflex (H-reflex) by TMS. None of the observed modulation occurred at the level of motor cortex. Finally, using the long-latency facilitation of the SOL H-reflex, we were able to confirm that the suppression of MEP at ISI = 40ms occurred at a premotoneuronal level. The data indicate that vestibular signals modulate corticospinal output to SOL at both premotoneuronal and motoneuronal levels during standing.

## Introduction

Balance control requires the integration of somatosensory, visual and vestibular information [1] and many supraspinal structures have been shown to be involved in this sensory integration [2]. Notably, the vestibular system is particularly important for maintaining body and head orientation in space [3]. One of the impacts of vestibular dysfunction on motor control is hypermetric postural responses [4, 5], observed after unilateral or bilateral vestibular loss, leading to balance and gait deficits. However, the underlying mechanisms of such defective responses are not entirely clear, mainly because the effects of vestibular afferents on corticospinal motor output has not been fully understood even in healthy subjects.

The motor cortex has also been implicated in the production of postural responses during standing or following perturbations [6–9] and evidence strongly indicates interactions with the vestibular system. In humans, stimulation of vestibular afferents could evoke potentials at cortical levels [10, 11]. fMRI studies further showed that 1) five different pathways could relay information from the vestibular nuclei to the parieto-insular vestibular cortex (PIVC) [12] and that, 2) after acute unilateral or bilateral vestibular lesions, changes in processing patterns of sensory modalities in the visual, somatosensory and auditory cortices were observed [13]. Recently, electrophysiological studies have investigated the nature of the vestibular influence on motor output and found that galvanic vestibular stimulation (GVS) could facilitate motor evoked potentials (MEPs) in arm muscles through the cervical interneuronal system [14]. Furthermore, Guzman-Guzman-Lopez et al. [15] demonstrated that caloric vestibular stimulation could modulate MEPs in the neck muscles. Their results suggested that the vestibular modulation of corticospinal control of the sterno-cleido-mastoid (SCM) likely occurred at cortical levels. Even though these studies strongly support interactions between vestibular and cortical systems in the control of arm and neck muscles during sitting or lying down, there are none during standing, or any other postural tasks. Hence, the nature of vestibulo-cortical interaction required for complex balance control remains unknown.

Several studies in animal models have described neuronal networks where vestibular and corticospinal systems can converge. Studies in cats and primates have revealed that many cortical areas receive information from the vestibular nuclei, including nuclei projecting to the spinal cord, such as the PIVC and the primary somatosensory cortex [16–18]. Other studies have shown convergence of the vestibular and corticospinal systems on spinal interneurons in the cat [19] and the macaque [20]. On the other hand, some reports have also demonstrated independent pathways to motoneurons from lateral vestibular nucleus and pyramidal tracts in the cat [21–24].

Thus, there appears to be several converging and separate pathways for vestibular and cortical signals to control motoneurons. This must provide the versatility necessary to learn and produce adequate anticipatory and compensatory postural responses for each movement. The aim of this study is thus to identify the nature, timing and localization of convergent signals from the vestibular and cortical systems during a simple postural task. Details about convergent pathways are necessary to understand the basic networks of postural control and their potential plasticity following a central nervous system (CNS) injury, such as vestibular dysfunction. Specifically, in the current study, we investigated the influence of the vestibular system on corticospinal output in Soleus muscle (SOL) during upright standing. We first determined the nature of this influence (facilitatory or inhibitory) on the corticospinal output and the delay at which the modulation occurred with respect to the vestibular responses. Second, we investigated whether these interactions occurred at motoneuronal, premotoneuronal or at the motor cortex levels. We expect the motor cortical output to be modulated by vestibular afferents both at the spinal cord level and in cortical areas. Parts of these results have been presented in abstract form [25].

## Methods

### Subjects

Twenty-five right-handed subjects (11 men and 14 women; mean age: 25.7 ± 4.6 years) participated in this study. Right-handed subjects were chosen based on evidence suggesting lateralization of vestibular function [26]. Handedness was confirmed using the Edinburgh test [27]. Participants received oral and written information about the experimental design and then gave their written consent to the study. The experimental protocol was approved by the local ethics research board of Centre for Interdisciplinary Research in Rehabilitation (CRIR#845–0613) and was in accordance with the Declaration of Helsinki. Subjects were excluded if they had: history of a brain injury or seizures; neurological or orthopedic disorder; family history of seizures; pregnancy; heart disease; cardiac pacemaker, or if they were taking medication that could have important effect on cortical and spinal excitability (e.g. epileptic or psychoactive drugs [28]).

### Experimental design

The experimental protocol comprised three parts. In Part A, fourteen subjects participated in 2 sessions of 2.5 hours in order to determine the nature and the timing of the modulation of MEP by GVS. In Part B, ten subjects took part in one session of 2.5 hours to determine whether the modulation could occur specifically at a cortical level, by assessing the effect of GVS on the short-latency cortical facilitation of the Hoffmann reflex (H-reflex). In Part C, 10 subjects took part in one session of 2.5 hours to determine whether modulation could occur at pre-motoneuronal level by assessing the effect of GVS on the long-latency cortical facilitation of the H-reflex. Three subjects took part in both Part A and Part C; 6 subjects took part in Parts B and C; and 1 subject took part in all three parts. Each part is described in detail below.

### Electromyographic recording

Electromyographic (EMG) activity was recorded from the right SOL, using bipolar surface electrode DE-2.1 (Delsys Bagnoli-8). This muscle was chosen based on its involvement in the production of postural responses at the ankle [29]. The electrodes were applied over the belly of the muscle in accordance with SENIAM recommendations (seniam.org; [30]). A reference electrode was positioned on the tuberosity of the right tibia. EMG signals were band-pass filtered (20–450 Hz), amplified (x100-1000) then digitized and sampled at 2 kHz to a computer using Micro1401 interface and Signal 4.07 software (Cambridge Electronic Design Ltd, Cambridge UK) for on-line and off-line analyses.

### Task

**Fig 1:**
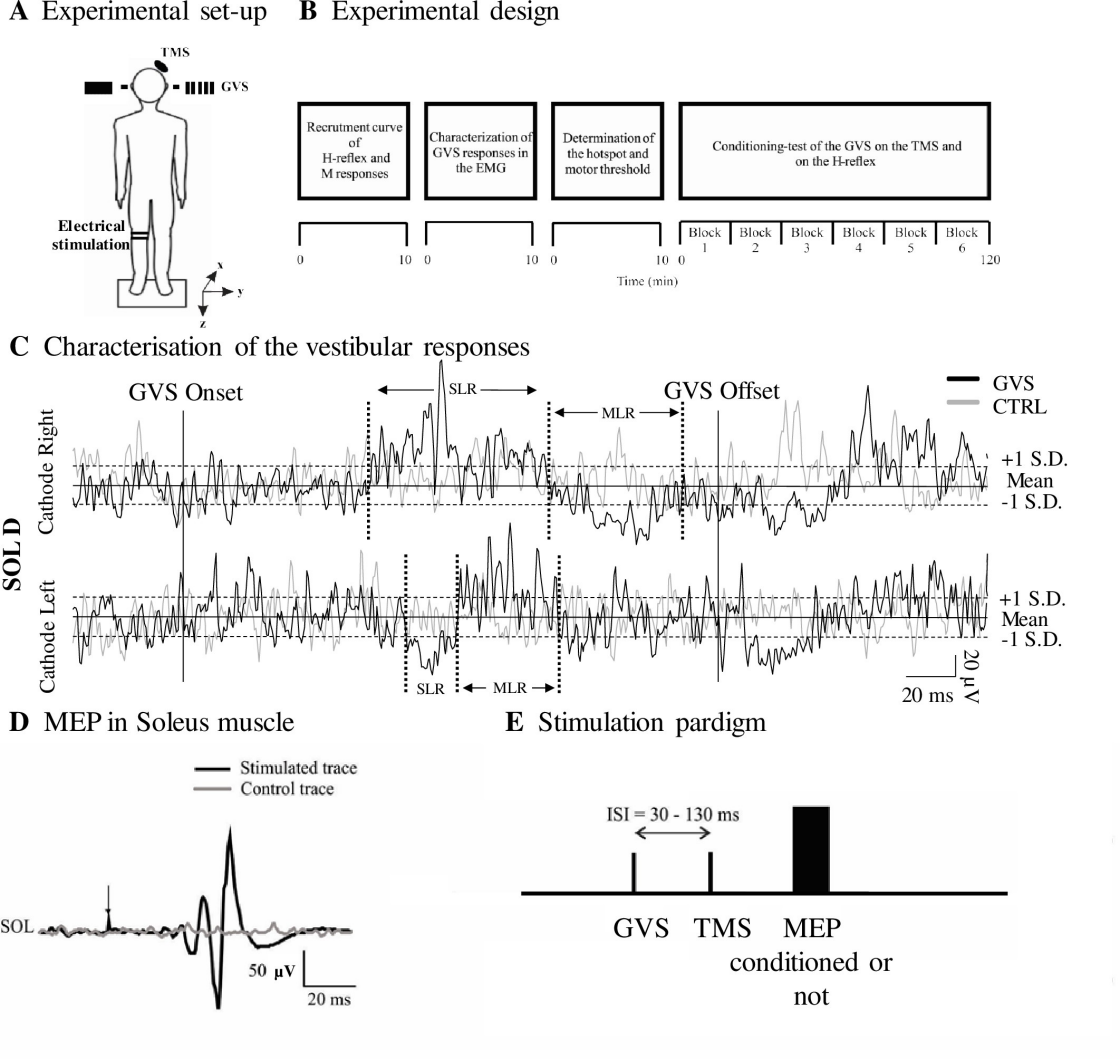
Overview of the experimental procedures. A. The schematic view of the experimental setup: Conditioning-test of the GVS on the MEP and on the H-reflex. The coil of the transcranial magnetic stimulation (TMS) was placed over the left primary motor cortex; The electrodes of the galvanic vestibular stimulation (GVS) were placed on the mastoid behind each ear; The H-reflex electrode was fastened to the right leg by a custom-made rectangular plate and straps. B. Experimental design. Time course of the experimental procedures during a session (2.5 hours). C. Data of one subject representing the effects of the bipolar galvanic vestibular stimulation (GVS) on the electromyographic (EMG) activity of the SOL muscles. The average rectified EMG responses of the right SOL with cathode right/anode left GVS (cathode behind the right ear) stimulation (30 trials at 3.5 mA) is shown in black, the control EMG, in grey. The two full vertical lines show the onset and offset of the 200 ms GVS pulse. The two dashed horizontal lines represent 1 SD above and below the mean background activity prior to the stimulation (solid horizontal line). D. Motor evoked potentials (MEP) induced by TMS in the right SOL (average of 10 stimuli). Grey trace: not stimulated; black trace: stimulated. E. Illustration of the stimulation paradigm where GVS and TMS are applied at different ISIs (30–130 ms) to MEP.

Subjects stood barefoot on a force platform (AMTI), kept their arms extended along the body, with their feet approximately 15 cm apart which corresponds to a comfortable distance for each subject but one that is smaller than their basic natural base of support in order to increase the instability of the task (Fig 1A). Subjects were asked to lean to the right and forward in order to induce a higher tonic contraction in the right SOL and facilitate the induction of MEP in the right SOL by increasing the motoneuronal excitability and reducing the motor threshold [31]. The level of SOL tonic contraction was fixed at 2–5% of the maximal M-wave (M_max_) quantified by a tibial nerve stimulation (TNS; see *SOL H-Reflex* section for M_max_ protocol).

Subjects were instructed to keep their head straight (face forward) during the acquisition so that confounding effects of head position were minimized [32–36]. Subjects were also instructed to keep their eyes closed during all acquisitions because evidence suggests that vision can modulate galvanic responses [37]. Throughout data collection, verbal feedback was given by the experimenter to ensure the head of the participant remained straight, the eyes closed, and the appropriate level of SOL contraction maintained. The level of SOL EMG (rectified and smoothed; 2^nd^ order, 1 Hz low pass Butterworth filter) was constantly monitored online on screen by the experimenter to remain between 2–5% M_max_.

### Galvanic vestibular stimulation

To apply GVS, the skin over the mastoids (behind the ears) was first rubbed with abrasive tape (3M Red Dot Trace Prep) prior to applying the electrodes coated with a gel to enhance conductivity (EC2® genuine grass electrode Cream). The electrodes were then fastened with tape, cotton padding was put over each electrode and a headband covered both electrodes to ensure a maximum contact between the electrodes and the skin. The impedance was measured with an ohmmeter and maintained at or below 1 kΩ to ensure minimal resistance to the current flow. The electrodes were connected to a constant current stimulator (model DS2, Digitimer, UK; maximal voltage of 100 V) delivering a 200 ms square-wave pulse at an intensity of 3.5 mA for all subjects. This intensity was chosen in order to induce postural responses that could be detected visually in all subjects without being too uncomfortable. Responses induced by GVS were recorded in the EMG of right SOL during standing (see Fig 1B). In order to characterize EMG responses to GVS, we applied 30 stimuli in a randomized manner over a period of 5 minutes with a minimum of 4 second intervals between stimuli. Although many studies have used 200–400 stimuli in order to obtain reliable vestibular responses to shorter stimulus durations (2-30ms) [38, 39], we used a relatively small number of stimuli (n = 30/polarity to characterize the EMG responses to GVS) as it was previously shown to be efficient to trigger vestibular responses [32, 40, 41] and in order to limit the duration of the experimental sessions.

In Part A, experiments were spanned over 2 sessions to assess the effect of the polarity of the GVS: In the first session, GVS was applied with the cathode right/anode left configuration and in the second session, GVS was applied with the anode right/cathode left. In Parts B and C, all data were collected in one session.

### Transcranial magnetic stimulation

Single-pulse TMS (Magstim Bistim2, Magstim Company Ltd., UK) was applied using a double-cone coil (70 mm outer diameter) over the leg area of the left primary motor cortex while the subject was standing. The optimal position (hotspot) for evoking MEP preferentially in the right SOL was determined. The hotspot was defined as the scalp position where the stimulus produced the largest motor evoked potential (MEP) amplitude in the right SOL at a given intensity [42–44]. Overall, this location was slightly to the left side of the vertex (mean of 1.2 cm left of the vertex). The hotspot was visualized on a computer through a neuronavigation system (Brainsight system; Rogue Research, Canada), by placing trackers both on the coil and on the subject. With this system, the coil was maintained at the same position throughout the experiment. The coil was held by an experimenter and positioned at around 45 degrees away from the midline over this hotspot to induce a current flow in an antero-medial direction in the targeted area [45]. The active motor threshold (aMT) was determined as the stimulus intensity at which 5 of 10 stimuli evoked a MEP bigger than 100 μV of amplitude in the contracted right SOL [31]. Stimulus intensity was set at 1.2 aMT for Part A and 0.95 aMT for Parts B and C.

### SOL H-reflex

The SOL H-reflex was evoked using TNS with a monopolar set-up: a half-ball metal cathode of 22 mm in diameter was placed in the popliteal fossa at an optimal position to induce the H-reflex, and a rectangular anode (8 cm x 15 cm) was placed on the anterior aspect of the knee. To ensure the appropriate placement of the electrodes, a stimulation high enough to elicit an H-reflex was applied and the electrode was moved around the popliteal fossa to determine the place where the amplitude of the H-reflex was the largest with a given stimulus intensity. Both electrodes were secured to the leg using a custom-made rectangular plate and straps. A 1 ms square-wave pulse (constant-current stimulator model DS7A, Digitimer, UK) was delivered through the electrodes. At the beginning of each session, a full H-reflex/M-Wave recruitment curve was recorded in standing position (single stimulation shown in Fig 1D). The stimulus intensity was increased in increments of 2 mA starting below H-reflex threshold up until maximal H-reflex response followed by increments of 10 mA to reach the M_max_.

For Part A, the targeted H-reflex was always on the ascending slope of the H-reflex recruitment curve [46, 47] and its size—measured as the peak-to-peak amplitude of the EMG signal—was then adjusted so that it was comparable to the peak-to-peak amplitude of the MEP in the right SOL (induced at 1.2aMT) [48]. In 8 subjects, it was possible to obtain comparable size of MEP and H-reflex within 5% of each other. However, in the 6 other subjects, MEP size remained lower than the H-reflex in SOL leading to larger gaps in amplitude. This difficulty to adjust the intensity of TNS and TMS to evoke test responses of similar sizes was documented previously [49]. As the effects of GVS on MEP and on H-reflex were not different between those 2 groups of subjects, their data was pooled together.

For Part B and C, the targeted H-reflex was also on the ascending slope of the H-reflex recruitment curve [46, 47] with a high-enough intensity to be accompanied by a M-wave equivalent to 5±2% of the M_max_. The amplitude of the M wave was monitored and constantly adjusted to remain within the targeted value for all conditions. In parts B and C, a MEP was not evoked by the TMS (0.95 aMT) and only the facilitation of the H-reflex amplitude with and without GVS application was assessed. To ensure that the activation of the tibial nerve was always similar in each condition, we monitored the M-wave.

### Part A—Effect of GVS on MEP

To investigate the modulation of corticospinal output by vestibular stimulation, a conditioning-test paradigm was used in which the conditioning stimulus consisted of the activation of vestibular afferents by the GVS (intensity 3.5 mA) and the test stimulus was the MEP induced by TMS (intensity 1.2 aMT). To determine if the interactions occurred at motoneuronal or pre-motoneuronal sites, the effects of GVS on MEP were first compared to the effects of GVS on the EMG background activity, induced when only the GVS is applied. A change in the area of the rectified EMG signal when the GVS is applied alone (compared to the area of the rectified EMG when no stimulation is applied) and that mimics the change in amplitude of the MEP would indicate a direct action of the GVS on the motor pool (a change in motoneuronal excitability) and thus a possible site for interaction with corticospinal volleys. Thus, when the effects of GVS were similar on background EMG area and on the MEP, this indicated that the change in motoneuronal excitability could explain the change in MEP amplitude (i.e. that the interaction occurred at the motoneuronal level).

Second, the effects of GVS on MEP was also compared to the effects of GVS on the SOL H-reflex because the latter is a good monitor of the overall excitability in spinal neuronal networks ending on the motor pool [47, 50]. If the effect on the H-reflex is similar to the effect on the background EMG and on MEP this would further suggest an action on the motor pool. If the effect of the H-reflex is different than the effect on MEP and/or on background EMG, that suggests a possible premotoneuronal effect of the GVS at the tested delay. The results of these comparisons indicate whether the vestibulocortical interaction occurred in motoneuronal or premotoneuronal networks.

A wide range of interstimulus intervals (ISI) was carefully chosen to enable observation of interactions between vestibular and cortical outputs at different levels of the neuraxis, based on the following documented latencies. The short-latency response (SLR) to GVS is expected to occur in the EMG around 60 ms after the onset of the stimulation and to last approximately 35 ms, and the medium-latency response (MLR) is expected to occur around 100 ms after the onset of the GVS and to last about 30–40 ms [32, 40, 51]. The long-latency responses (LLR) is expected to start shortly after the end of the MLR at around 140–150 ms. Because the latency of MEP in the SOL is about 30–35 ms [52, 53], GVS needed to be applied prior to the TMS in order to induce either SLR, the MLR or the LLR simultaneously to the MEP response (see Fig 1D). GVS was applied prior to the TMS at ISI = 30, 40, 50, 70, 90, 100, 110 and 130 ms. During data collection (see Fig 1B), two ISI (ISI#1 and ISI#2) were tested per block of acquisition. For each block of acquisition, 10 trials of each of the following conditions were randomly alternated: no stimulation, GVS alone, TMS alone, H-reflex alone, GVS and TMS combined for ISI#1, GVS and H-reflex combined for ISI#1, GVS and TMS combined for ISI#2, and GVS and H-reflex combined for ISI#2. At least 4-second intervals separated each trial. In total 6 blocks of acquisition were performed per session. Pauses were taken in the middle of each block and between each block to prevent muscle fatigue. More pauses could be taken if requested by the subject.

Although these ISIs are of long duration, the responses discussed do not correspond to off responses, which are induced by the offset of the GVS stimulus pulse [54]. We stimulated from 60 ms after GVS onset (ISI at 30ms) to 160 ms (ISI at 130 ms). Since the duration of the GVS pulse is 200 ms, we stimulated within the duration of the GVS pulse. The responses are thus due to continuous stimulation and not stimulus offset.

### Effect of GVS on the TMS-evoked short-latency and long-latency facilitation of the right SOL H-reflex

**Fig 2:**
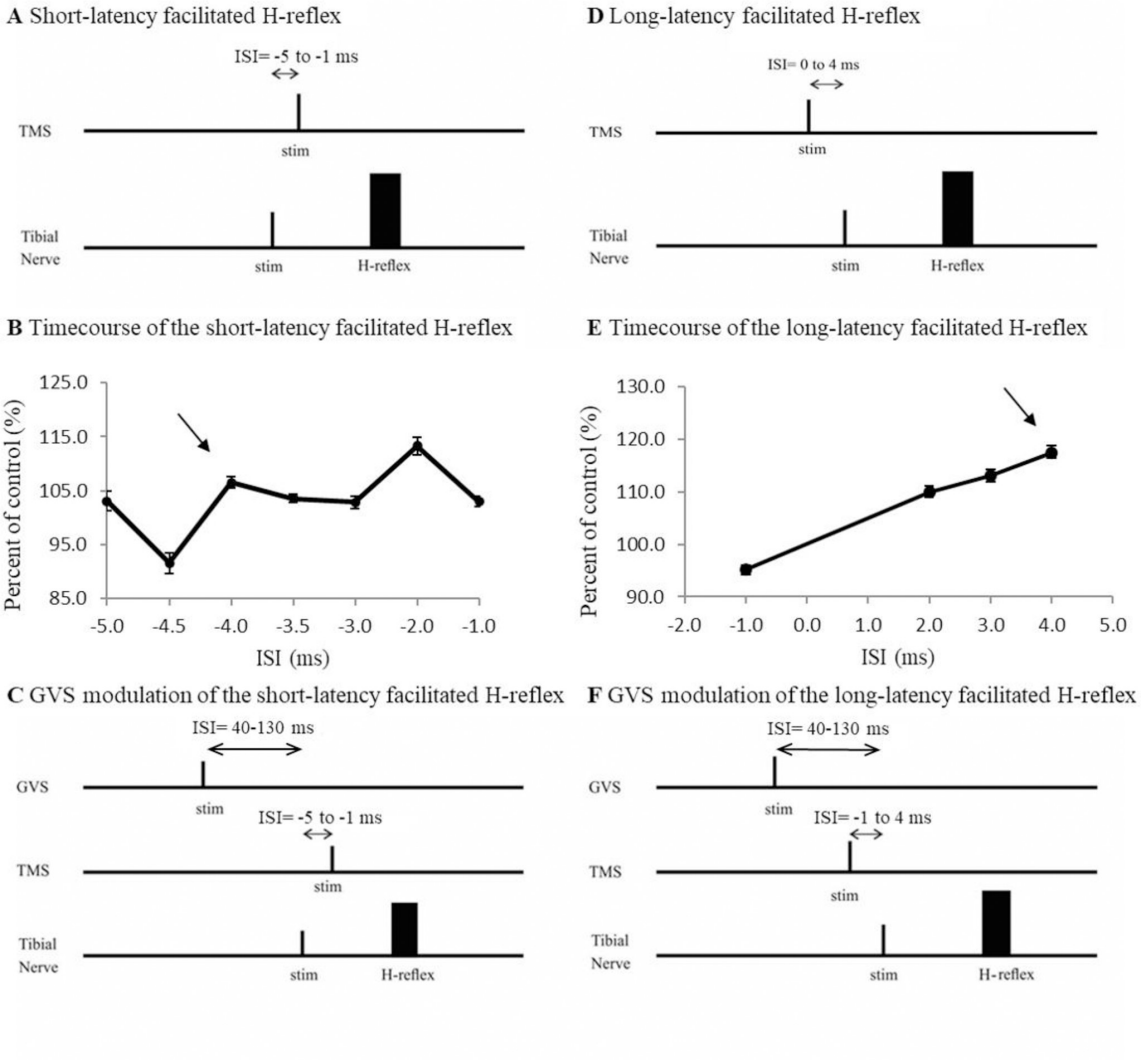
Short-Latency and long-latency facilitation paradigm. A. Illustration of the short-latency facilitation where TMS pulse follows the application of the tibial nerve (ISI = -5 to -1 ms) that will trigger an H-Reflex. B. This paradigm induces a facilitation of the H-reflex amplitude and the earliest delay at which the facilitation occurred (arrow) was determined and used to assess influence of GVS on cortical neurons. C. To assess the effect of GVS on the facilitated H-reflex, GVS was applied 40 and 130 ms (cathode right/anode left) prior to the tibial nerve. D. Illustration of the long-latency facilitation where TMS pulse follows the application of the tibial nerve (only ISI -1, 2, 3 and 4 ms shown in this subject) that will trigger an H-Reflex. E. This paradigm induces a facilitation of the H-reflex amplitude and the delay at which the largest facilitation occurred (arrow) was determined and used to assess influence of GVS on corticospinal tract. F. To assess the effect of GVS on the facilitated H-reflex, GVS was applied 40 and 130 ms (cathode right/anode left) prior to the tibial nerve.

To investigate whether the modulation of corticospinal output by vestibular stimulation occurred at cortical level, the effect of GVS on the TMS-evoked facilitation of the H-reflex was assessed. Nielsen and colleagues [55–58] have demonstrated that the H-reflex was facilitated by TMS at very short latencies. They used negative conditioning-test intervals (-5 to -1ms, by steps of 0.5 ms), where the conditioning stimulus (TMS) was applied after the test stimulation (H-reflex), to highlight the onset of this facilitation. They further demonstrated that the initial part of the facilitation (called here short-latency facilitation) was due to activation of cortical cells with monosynaptic projections to the motoneurons. Changes in the amplitude of the short-latency facilitation reflect changes at a cortical level if the corticospinal fibers are not directly activated by TMS (i.e., subthreshold for eliciting a D-wave), and motoneuronal excitability remains unchanged. They also showed that the later part of this facilitation (called here long-latency facilitation) was due to mixed activation of the cortical cells with monosynaptic projections and other cortical cells with non-monosynaptic projections to the motoneurons. Thus, changes in the amplitude of the long-latency facilitation reflects changes along the corticospinal tract at cortical and premotoneuronal levels, but not strictly at cortical levels. Fig 2A and Fig 2D show the stimulation protocol used to elicit a TMS-evoked short and long facilitation of the H-reflex respectively. Fig 2B and Fig 2E show the time course of both facilitations in a representative subject and the conditioning-test interval that was chosen to assess the modulation of GVS on the short- and long-latency facilitation of the H-reflex. In the following two sections, the methodology to assess the effects of GVS on the short-latency and the long-latency facilitations is described.

### Part B—Effect of GVS on the TMS-evoked short-latency facilitation of the right SOL H-reflex could reflect convergence at cortical level

As detailed above, the short-latency facilitation of H-reflex is thought to reflect the direct connections between cortical cells and motoneurons [55, 58]. Hence, a change occurring in the amplitude of the short-latency facilitation by conditioning of GVS can be attributed to changes in the excitability of cortical neurons with monosynaptic projections to the spinal motoneurons.

To induce the short-latency facilitation of the H-reflex, a protocol similar to that described in previous studies [55, 56, 58] was used. Stimulation of the right tibial nerve to evoke a SOL H-reflex (M wave = 5±2% M_max_) and TMS (0.95 aMT) over the SOL area of motor cortex were applied at negative conditioning-test intervals of -5, -4.5, -4, -3.5, -3, -2 and -1 ms in 10 subjects. The first step was to determine the earliest conditioning-test interval where H-reflex was facilitated (>5%), in order to be sure that only cortical cells with monosynaptic projections to the motoneurons were assessed (Fig 2B). The expression ‘facilitated H-reflex’ will be used to refer to the result of the short-latency facilitation of the H-reflex in the rest of this manuscript. Thereafter, GVS was applied prior to the TNS at the delays where MEP was significantly facilitated or suppressed in Part A (Fig 2C). Based on previous studies [59], the latency of the SOL H-reflex is between 30–35 ms and is similar to the latency of the MEP in SOL. Because the test response (MEP or H-reflex) occurs at the same latency we used the same ISI between GVS and the TMS as in Part A, and between GVS and the TNS in Part B.

Fig 2C shows the stimulating protocol used. GVS was applied prior to the TNS to determine if the amplitude of the facilitated H-reflex would be changed by the GVS at ISIs that significantly facilitated or suppressed the MEP, either at 40 or 130 ms with cathode right/anode left configuration. Ten repetitions of each of the following conditions were randomly alternated: no stimulation; TMS alone; H-reflex alone; GVS alone; facilitation of the H-reflex by the TMS (TMS+H); GVS preceding TMS+H at targeted delays. In each recording the short-latency facilitation of the H-reflex by TMS was retested to confirm the conditions were similar: if the H-reflex could no longer be facilitated by the TMS during a given recording with GVS, the data collected were discarded. Overall, 2 recordings had to be discarded in 2 different participants.

### Part C—Effect of GVS on the TMS-evoked long-latency facilitation of the right SOL H-reflex reflects convergence on spinal interneurons (premotoneuronal level)

As mentioned above, the long-latency facilitation of the SOL H-reflex is thought to reflect the excitability in oligosynaptic as well as monosynaptic corticospinal pathways [55–58]. As such a change in the amplitude of the long-latency facilitation of the SOL H-reflex by conditioning of GVS that is not accompanied by a similar change in the short-latency facilitation would indicate that the convergence occurs at a premotoneuronal level. A similar protocol as in Part B was used to induce the long-latency facilitation but with different conditioning-test intervals. Indeed, in 10 subjects, the TMS (0.95 aMT) was applied at conditioning test-intervals of -1, 1, 2, 3 and 4 ms with respect to the tibial nerve. Here, the delay where the H-reflex was the most facilitated was chosen. GVS was then applied prior to the tibial nerve stimuli at delays where MEP was significantly facilitated or suppressed in Part A (see Fig 2F).

### Data analysis

#### EMG responses to GVS

GVS evoked vestibular responses in the right SOL. The EMG signal was rectified and averaged for each polarity (cathode right/anode left, or anode right/cathode left configurations). From the averaged signal, it was possible to clearly determine the latency, amplitude and duration of EMG responses to GVS. The onset of the SLR was determined as the first time point after the delivery of the GVS when the EMG either dropped below or rose above 1 standard deviation (SD) of the control trials (expected latency around 60 ms) and the duration of this response had to be at least 5 ms. The offset of the SLR was measured at the time when the EMG crossed back the mean ± 1 SD. To identify vestibular responses, the mean background EMG and SD were calculated on the control trial (trial without stimulation) over the duration of the stimulation pulse (200ms); see Fig 1C. The same procedures were used to determine the onset and offset of the MLR (expected latency around 100 ms). The duration of the SLR and the MLR was defined as the time window from the onset to the offset of each response. To quantify the amplitude of the responses, the area under the SLR and MLR were calculated, and it was divided by the area EMG taken over the same duration of time in control condition. The result was then multiplied by 100, as shown in the following formula:

$$\%\;\mathrm{Inhibition}\;\mathrm{or}\;\mathrm{Facilitation}=\frac{EMG\;\mathrm{area}\;\mathrm{of}\;GVS\;\mathrm{condition}}{EMG\;\mathrm{area}\;\mathrm{of}\;\mathrm{control}\;\mathrm{condition}}\times 100$$

#### Conditioning of the MEP, of the H-reflex and of the background EMG by GVS

For each ISI, peak-to-peak amplitudes of the 10 conditioned and 10 unconditioned unrectified MEP were measured within a 30 ms window (between 30 and 60 ms after TMS application) for each trial. The averaged amplitude of the 10 conditioned MEP was then expressed relative to the averaged amplitude of the 10 unconditioned MEP to determine the percentage of facilitation or suppression induced by GVS. The following formula was used:

$$\%\;\mathrm{Inhibition}\;\mathrm{or}\;\mathrm{facilitation}=\frac{\mathrm{mean}\;\mathrm{amplitude}\;\mathrm{of}\;\mathrm{conditioned}\;MEP}{\mathrm{mean}\;\mathrm{amplitude}\;\mathrm{of}\;\mathrm{unconditioned}\;MEP}\times 100$$

The same procedures and formulas as described above for MEP were used to determine the effect of GVS on the H-reflex (peak-to-peak amplitude) and on the short-latency and long-latency facilitation of H-reflex (peak-to-peak amplitude). To assess the effect of GVS on background EMG, the area under the curve was quantified within the same 30 ms window corresponding to the targeted ISI (i.e. between 30 and 60 ms after what would have been a TMS pulse) in trials when GVS was applied alone (n = 10). Facilitation or suppression of EMG was calculated by dividing the area of the EMG response occurring when GVS was applied alone by the area of the EMG over the same duration when no stimulation is present (in control trials n = 10) and multiplying by 100. The response detected were used to enable direct comparison of the effect of GVS on MEP, on H-reflex and on background EMG in similar conditions (task, number of GVS pulses applied). For parts B and C, the mean background EMG level was compared between the different conditions and were calculated on the stimulated trial just prior to the application of the GVS/TMS or TNS pulse, over 200ms. Furthermore, we verified that the conditioning subthreshold TMS pulse by itself did not evoke a MEP. Indeed, to confirm that the conditioning TMS stimulus intensity was subthreshold, the mean background EMG level was compared to the mean EMG level in a 30ms window -between 30 and 60 ms- after the subthreshold TMS pulse was applied alone.

### Statistics

First, the normality of the variables (or difference of variables) was assessed using the Shapiro-Wilk test, which is the best one for small samples. This allowed us to choose between parametric and non-parametric tests for a given analysis as stated in the results for each analysis. If the normality was assumed (Shapiro-Wilk test not significant), to determine the effect of GVS on MEP, a Student’s one-sample t-test for comparison of MEP amplitude to 100% was conducted at each ISI. When significant, paired t-tests were conducted to compare the effect of GVS on MEP, EMG and H-reflex for SOL. To determine if the amplitude of the H-reflex was facilitated by the TMS, paired t-tests were conducted. To assess whether the amplitude of the facilitated H-reflex was changed by GVS, one-sample t-tests were performed. In part B and part C, modulation in the M-wave and background EMG between the conditions was assessed using repeated measures ANOVA tests. Non-parametric assessments (Wilcoxon signed rank test) were performed if variables (for one-sample t-tests) or differences (for paired t-tests) were not normally distributed. The non-parametric test (Related-Samples Friedman's Two-Way Analysis of Variance by Ranks) was performed if variables for repeated measures ANOVA were not normally distributed.

All values are expressed as mean ± SEM. Cohen’s *d* effect sizes for parametric analyses and r for non-parametric analyses were computed. Statistical significance was set α = 0.05. All analyses were conducting using SPSS version 20.0 (SPSS Inc, USA) for Windows (Microsoft Corporation, USA).

## Results

### Part A—The effect of GVS on the right SOL MEP

Characterization of EMG responses in SOL induced by GVS was done using an average of 30 stimuli (see Fig 1C; details in Table 1). EMG responses induced by cathode right/anode left GVS were observed in 11/14 participants. On average, the SLR consisted of a suppression of SOL EMG (81.73 ± 10.67%; 6 subjects with a suppression, 1 subject with a facilitation) at a mean latency of 68 ± 3 ms and the MLR consisted of a facilitation of SOL EMG (136.26 ± 8.94%; 8 subjects with a facilitation; 1 subject with a suppression) at a latency of 101 ± 4 ms. Anode right/cathode left GVS induced a SLR that was variable between subjects (3/6 subjects with a facilitation, 3/6 with a suppression) at a latency of 76 ± 4 ms and a MLR that was mostly facilitatory (5/8 subjects with a facilitation, 3/8 with a suppression) at a latency of 106 ± 5 ms. TMS was applied at the hotspot for each subject and the mean aMT for Part A was 58 ± 15% of the maximal stimulation output. TMS at 1.2aMT induced MEP in the right SOL of all subjects with a mean latency of 34 ± 1 ms and a peak-to-peak amplitude of 15.77 ± 1.90% of M_max_.

**Table 1:** Characteristics of responses induced by the bipolar galvanic vestibular stimulation (GVS) in the right SOL.

	Cathode right/anode left GVS						Cathode left/anode right GVS					
	SLR			MLR			SLR			MLR		
Subjects	Latency (ms)	Amplitude %	Duration (ms)	Latency (ms)	Amplitude %	Duration (ms)	Latency (ms)	Amplitude %	Duration (ms)	Latency (ms)	Amplitude %	Duration (ms)
1	71	74.73	24	96	133.15	17	58	48.40	43	102	148.91	16
2	-	-	-	-	-	-	74	149.16	10	90	75.99	18
3	65	82.01	24	98	141.37	19	86	143.65	36	127	65.68	20
5	69	64.63	8	93	146.11	15	77	73.45	9	94	136.59	33
7	-	-	-	104	154.34	20	-	-	-	-	-	-
8	-	-	-	-	-	-	-	-	-	98	171.89	20
9	65	63.55	14	93	154.25	22	75	131.99	37	127	72.44	8
10	83	64.79	13	114	151.65	10	83	66.60	14	103	128.47	31
12	57	78.89	20	81	145.38	32	-	-	-	-	-	-
13	-	-	-	104	126.57	18	-	-	-	107	131.61	13
14	68	143.5	23	126	73.50	32	-	-	-	-		-
Mean	68	81.73	18	101	136.26	21	76	102.21	25	106	116.45	20
SEM	3	10.67	2	4	8.94	2	4	18.07	6	5	14.05	3

The latency, amplitude and duration of the short latency response (SLR) and medium latency response (MLR) are reported. Both polarities are displayed. Data presented for 11/14 subjects; 3 subjects did not show any responses to GVS for either polarity in right SOL muscle.

**Fig 3:**
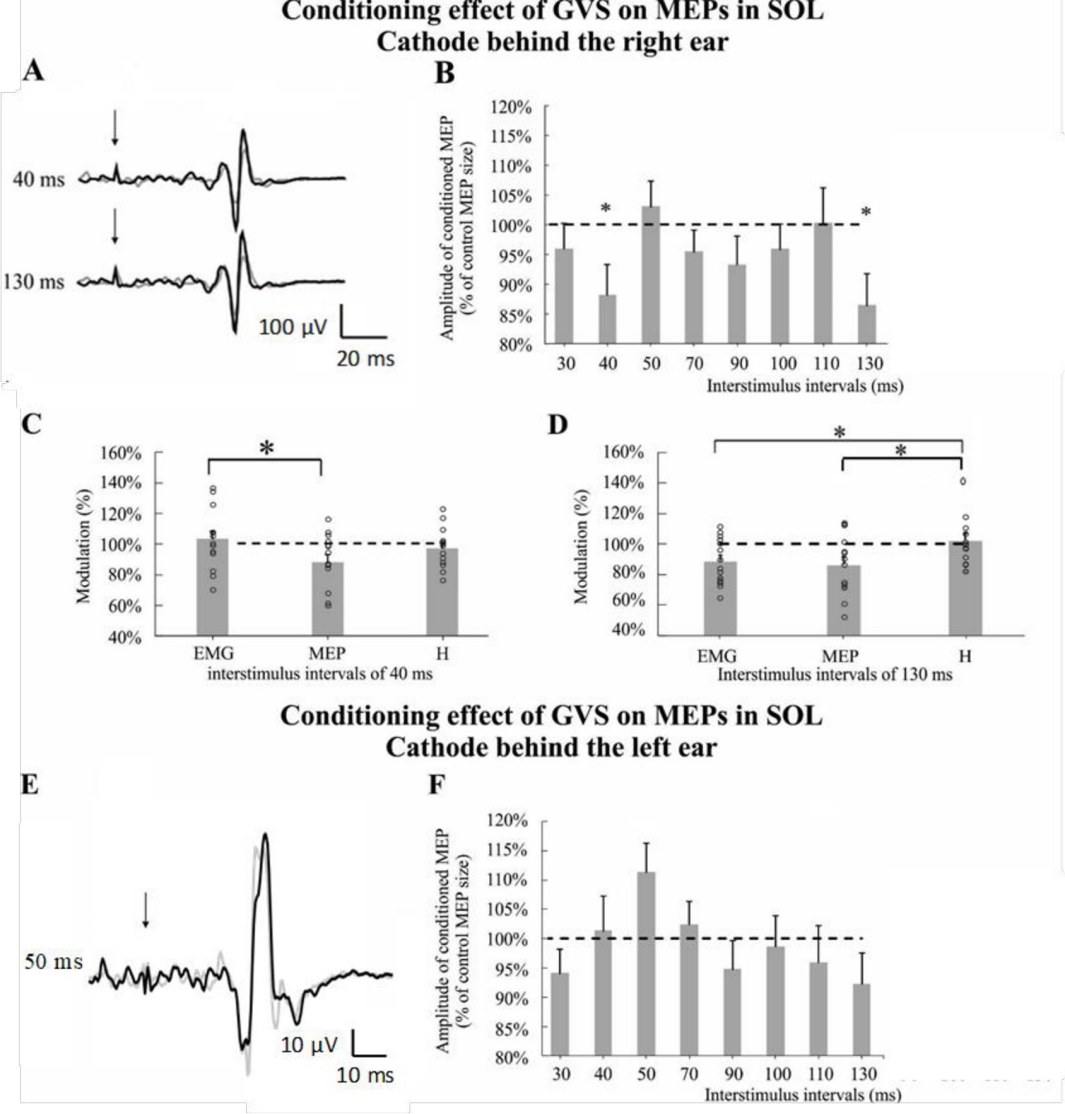
Modulation of motor evoked potential (MEP) in the right Soleus muscle (SOL) by galvanic vestibular stimulation (GVS). A and E. Sample EMG traces of the average MEP (n = 10) of a representative subject illustrating the conditioning effects of GVS for four different interstimulus intervals (ISIs = 0, 40, 130 and 50 ms); control MEP (black) and conditioned MEP (grey). B and F. Timing of the effect of bipolar GVS on the MEP. The histogram bars correspond to the mean effect observed for all the subjects (n = 14). The dashed black line corresponds to the control MEP size. C and D. Significant effect of GVS on MEP in SOL compared to the effect of GVS on the H-reflex and background EMG activity in SOL for the same interstimulus interval (40 and 130 ms). The histogram bars correspond to the mean effect observed for all the subjects (n = 14). Circles represent data from each subject. Error bars represent the standard error of the mean. *p<0.05.

Fig 3A–3D shows the effect of cathode right/anode left GVS on MEP. In Fig 3A, a decrease in MEP amplitude is observed at ISI = 40 and 130 ms for one representative subject. The data collected from the group of subjects (Fig 3B) also shows a significant decrease of MEP for ISI = 40 ms (88.15 ± 5.07%; Confident interval (CI): [77.2; 99.1]; one-sample t-test t(13) = 2.336, p = 0.036; Cohen’s d = 0.624) and ISI = 130 ms (86.42 ± 5.26%; CI: [75.1; 97.8]; one-sample t-test t(13) = 2.580, p = 0.023; Cohen’s d = 0.689). To determine whether changes were occurring at a motoneuronal level, the effect of GVS on the MEP was compared to the effect of GVS on the background EMG and on the H-reflex for the same ISI (Fig 3C and 3D). The H-reflex was assessed by monopolar stimulation of the tibial nerve (see Methods) and the mean peak-to-peak amplitude was 21.4 ± 3.1% of M_max_. At ISI = 40 ms (Fig 3C), GVS induced a suppression of the MEP, but had no effect on the amplitude of the EMG area. This difference was significant (88.15 ± 5.07% for MEP; 103.45 ± 5.22% for EMG; CI on difference: [5.2%; 25.4%]; paired t-test t (13) = 3.274, p = 0.006; Cohen’s d = 0.875). The effect of GVS on MEP was not statistically different than on the H-reflex amplitude (88.15 ± 5.07% for MEP; 97.33 ± 3.49% for H-reflex; CI on difference: [-3.2%; 21.5%]; paired t-test t(13) = 1.604, p = 0.133; Cohen’s d = 0.429) and no significant difference was observed between GVS modulation of the background EMG and of the H-reflex amplitude (103.45 ± 5.22% for EMG; 97.33 ± 3.49% for H-reflex; CI on difference: [-4.8%; 17.0%]; paired t-test t(13) = 1.214, p = 0.246; Cohen’s d = 0.325). At ISI = 130 ms (Fig 3D), GVS evoked a suppression of both MEP and the amplitude of the EMG area (86.42 ± 5.26% for MEP; 88.70 ± 4.02% for EMG; CI on difference: [-14.3%; 9.7%]; paired t-test t(13) = 0.409, p = 0.689; Cohen’s d = 0.109), but had no effect on the H-reflex (102.28 ± 4.98%). A comparison between the GVS effects on the MEP and H-reflex amplitude showed a significant decrease for the MEP (86.42 ± 5.26% for MEP; 102.28 ± 4.98% for H-reflex; CI on difference: [2.9%; 28.9%]; paired t-test t(13) = 2.637, p = 0.021; Cohen’s d = 0.705). A significant difference was also observed between the GVS modulation of the background EMG and of the H-reflex amplitude (88.70 ± 4.02% for EMG; 102.28 ± 4.98% for H-reflex; CI on difference: [3.4%; 23.8%]; paired t-test t(13) = 2.887, p = 0.013; Cohen’s d = 0.772). Thus, cathodal GVS induced a MEP inhibition at 40 and 130 ms. Whereas GVS did not change the background EMG at 40 ms, it decreased it at 130 ms. GVS did not have a significant effect on H-reflex at either ISI.

In Fig 3E and 3F, anode right/cathode left GVS only triggers a facilitation (trend) of the MEP at ISI = 50 ms (111.35 ± 4.95%; standardized Wilcoxon = 1.789, p = 0.074; r = 0.478), however it was not statistically significant. The effect of the GVS polarity (side of the cathode) was examined for each ISI separately (not shown). No significant effect of the polarity of the GVS (anode right/cathode left vs cathode right/anode left configuration) was found on the MEP amplitude and on the EMG area for the same ISI.

### Part B—The effect of GVS on short-latency facilitation of the H-reflex

**Fig 4:**
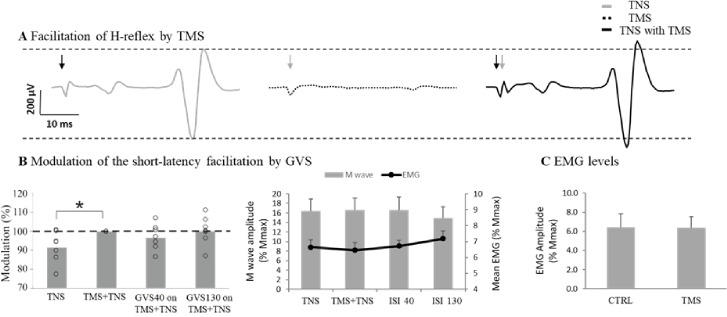
Modulation of the short-latency TMS-facilitated H-reflex in the Soleus muscle (SOL) by galvanic vestibular stimulation (GVS). A. Short-latency facilitation of the H-reflex by TMS. Left trace (grey) is H-reflex in the Soleus of one standing subject (N = 10 sweeps). The dotted trace is the SOL EMG when only TMS is applied. The right trace (black) is SOL H-reflex when it was conditioned by the TMS at ISI = -3ms are superimposed, which leads to an increased H-reflex amplitude. B. Modulation of the facilitated H-reflex by GVS for the group. In the left graph, the 1^st^ and 2^nd^ histograms show a significant facilitation of the SOL H-reflex by TMS. The 3^rd^ and 4^th^ histograms show the effects of GVS at 2 different ISIs. Overall, there was no significant effect of GVS on the short-latency facilitated H-reflex. Mean value for each subject (circle) were also superimposed on the histogram bars. In the right graph, control M wave (bars; Y axis on the left) and background EMG level (line; Y axis on the right) are displayed for the group. C. Comparison between the mean background EMG level and the mean EMG level following application of subthreshold TMS alone. No significant effect is observed. * = p<0.05.

Using the short-latency facilitation paradigm shown in Fig 2A, we assessed the difference in amplitude of H-reflex with and without conditioning by the TMS at short latencies. The mean aMT for all subjects was 54±11% of maximum stimulator output. Fig 4A shows the difference in the amplitude of the soleus H-reflex alone (left trace) and when it is conditioned by TMS at conditioning-test interval of -3 ms (right trace) in one representative subject during quiet standing. The middle trace shows the mean EMG level when TMS was applied alone. Each trace represents an average of 10 stimuli. In this subject, the amplitude of the H-reflex increased to 108% of control value when it was conditioned by the TMS, and the TMS itself did not evoke a MEP. Fig 4B shows the effects for the group. In the left graph, the H-reflex was significantly facilitated by the TMS in 7 subjects (H-reflex = 21.013±2.160% M_max_; TMS+H = 23.018±2.468% M_max_, difference CI: [0.011; 0.119]; paired t-test t(6) = 2.961, p = 0.025; Cohen’s d = 1.12).

In the preceding Part A, no significant effect of GVS on the H-reflex was found at ISI 40 and 130 ms (97.33% and 102.28% of unconditioned H control at 40 and 130 ms respectively). Here, the effect of GVS on the facilitated H-reflex showed a large variability between participants and, overall, the short-latency facilitation induced in SOL H-reflex remained unchanged by cathode right/anode left GVS at 40 ms (mean = 97.17±2,30% of control; Paired t-test t(6) = 1.334, p = 0.231; Cohen’s d = 0.5) and at 130 ms (mean = 102.39±3.50%; CI:[90.3%; 102.9%]; one sample t-test t(6) = 0.026, p = 0.980; Cohen’s d = 0.0097). In the right graph, the mean amplitude of M-wave and background EMG is shown. No statistical difference was observed for either the M-wave or the EMG between conditions. (Related-Samples Friedman's Two-Way Analysis of Variance by Ranks, p = 0.522 and p = 0.753 respectively). Also, as a control to verify that the conditioning subthreshold TMS pulse by itself did not evoke a MEP, the background EMG level was compared to the mean EMG level following the application of subthreshold TMS (see Methods). As shown in Fig 4C, this difference was not significant (6.41 ± 1.41%Mmax for CTRL; 6.38 ± 1.18%Mmax for TMS; CI on difference: [-0.71; 0.77]; paired t-test t(7) = 0.097, p = 0.925; Cohen’s d = 0.034)

### Part C—The effect of GVS on long-latency facilitation of the H-reflex

**Fig 5:**
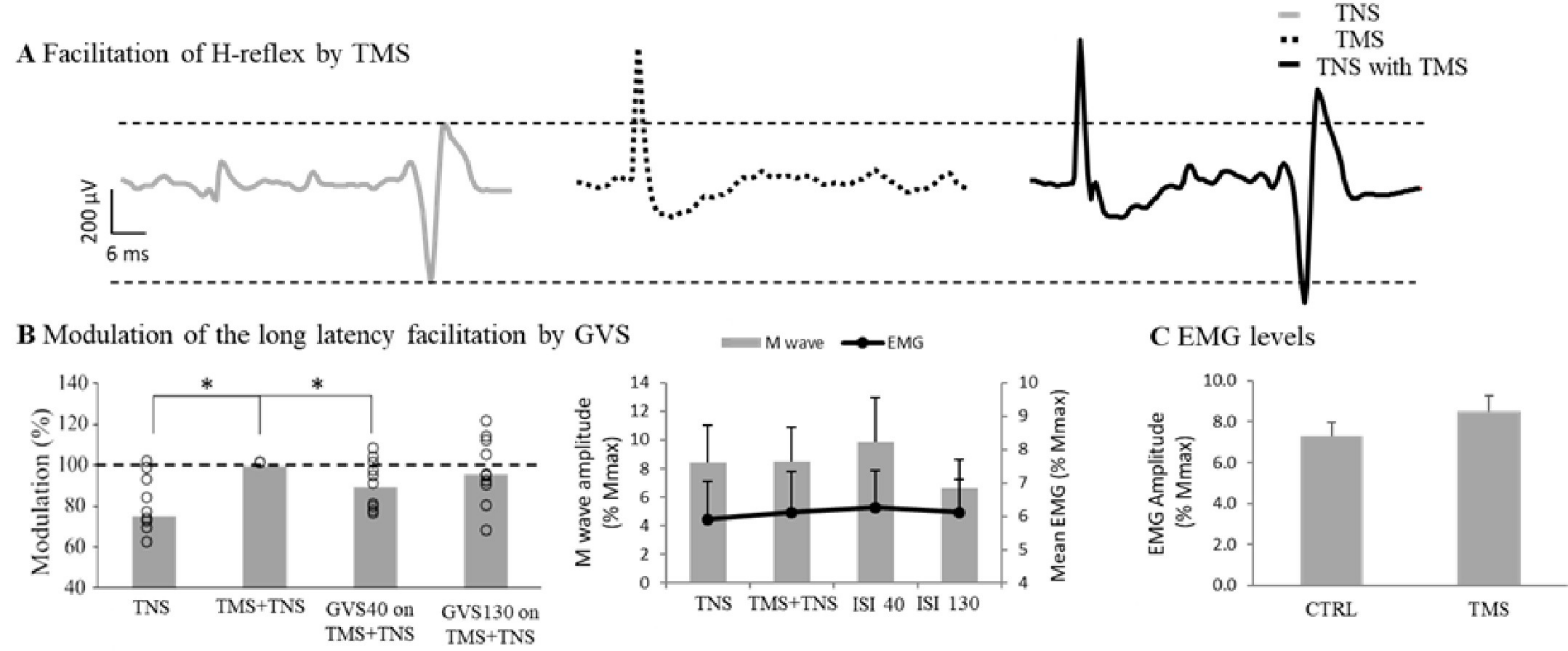
Modulation of the long-latency TMS-facilitated H-reflex in the Soleus muscle (SOL) by galvanic vestibular stimulation (GVS). A. Late facilitation of the H-reflex by TMS. Left trace (grey) shows H-reflex in the Soleus of one standing subject (N = 10 sweeps). Middle trace (dotted) shows the SOL EMG when only TMS is applied. Right trace (black) shows the SOL H-reflex when it was conditioned by the TMS at ISI = 2ms, which leads to an increase in the H-reflex amplitude. B. Modulation of the facilitated H-reflex by GVS for the group. In the left graph, histograms representing effect of GVS on the facilitated H-reflex for the group. Control amplitude (dashed line) was that of the facilitated H-reflex (TMS on H). Mean value for each subject were also superimposed on the histogram bars (circles). In the right graph, control M wave (bars; Y axis on the left) and background EMG level (line; Y axis on the right) are displayed for the group. C. Comparison between the mean background EMG level and the mean EMG level following application of subthreshold TMS alone. No significant effect is observed. * = p<0.05.

Fig 5A shows the difference in the amplitude of the soleus H-reflex alone (left trace) and when it is conditioned with TMS at a conditioning-test interval of 2 ms (right trace) in one representative subject during quiet standing. In Fig 5A, the amplitude of the H-reflex in this subject increased to 127% when conditioned by TMS. Fig 5B illustrates the effects for the group. The mean aMT for all subjects was 55± 11% of maximum stimulator output. In the left graph (cathode right/anode left GVS) the H-reflex was significantly facilitated by the TMS in all subjects (H-reflex = 33.194 ± 6.121% M_max_; TMS-H = 52.577±8.985% M_max_; CI on difference:[0.138; 0.650]; paired t-test t(9) = 3.482, p = 0.007; Cohen’s d = 1.1). This facilitation (dashed line) was significantly decreased when GVS was applied at an ISI of 40 ms (GVS-40ms = 89.7±3.7% of control; CI:[81.2; 98.2]; one-sample t-test t(9) = 2.753, p = 0.022, Cohen’s d = 0.87) but not at 130 ms (mean = 95.97±5.2% of control; CI:[84.3; 107.6]; one-sample t-test t(9) = 0.780, p = 0.455; Cohen’s d = 0.25). In the right graph, the mean amplitude of M-wave and background EMG is shown. No statistical difference was observed for either the M-wave (Related-Samples Friedman's Two-Way Analysis of Variance by Ranks, p = 0.154) or the EMG (RM ANOVA, p = 0.635) between conditions. Furthermore, to verify that the conditioning subthreshold TMS pulse did not evoke a MEP by itself, the mean background EMG and the mean EMG level following the application of subthreshold TMS alone were compared. As shown in Fig 5C, this difference was not significant (6.82 ± 0.70%Mmax for CTRL; 6.84 ± 0.76%Mmax for TMS; CI on difference: [-0.58; 0.0.54]; Standardized Wilcoxon on Difference = 0.560, p = 0.575; r = 0.198).

## Discussion

We have used electrophysiological methods to determine the timing and nature of the modulation exerted by the vestibular system on corticospinal output to the right SOL during standing, a task requiring balance control. Overall, the major findings of this study are: 1) At latencies corresponding to SLR and beginning of MLR (ISI = 40ms), the vestibular output suppresses the corticospinal activity on the side of the cathode; 2) At latencies corresponding to the end of the MLR and beginning of LLR (ISI = 130 ms), the vestibular output suppresses corticospinal activity on the side of the cathode; 3) the vestibular output did not modify the amplitude of the SOL H-reflex at either ISI = 40 ms or 130 ms; 4) The suppression of corticospinal activity observed at 40 ms on the side of the cathode occurs at a pre-motoneuronal level; 5) The suppression occurring on the side of the cathode at 130 ms occurs at the motoneuronal level; 6) None of the interactions observed occurred at the level of the motor cortex.

### Effects of vestibular stimulation on MEP, EMG and H-reflex

The responses with the cathode right/anode left configuration of GVS (N = 30 stimulation) were variable, but the SLR mainly consisted of a suppression of EMG and the MLR was a facilitation of the EMG. We then assessed the modulation of MEP by GVS in Part A with a smaller number of sweeps (n = 10) than in the characterization of EMG responses to GVS, so that a full-blown response is not elicited in the EMG. In that way if a change is observed in the MEP, but not in EMG or H-reflex, that would suggest that the change is not simply due to changes in motoneuronal excitability. Using this approach, we observed a decreased MEP amplitude at 40 ms (corresponding to the SLR) as well as at 130 ms (corresponding to the end of the MLR).

To delineate the sites of interaction, additional analysis and experiments were performed. If MEP modulation by GVS occurred at a motoneuronal level, one would expect the GVS alone to modify motoneuronal excitability as monitored by the background EMG area. However, if GVS alone did not affect EMG area then one could infer that interaction occurred before reaching the motor pools i.e. at a premotoneuronal level (either spinal or supraspinal). A significant decrease of the right SOL MEP amplitude by cathode right/anode left GVS occurred at 40 ms without changes in EMG area. This strongly suggests that the vestibular system was able to decrease the corticospinal output at a premotoneuronal level at this delay. On the other hand, GVS applied at ISI = 130 ms in a cathode right/anode left configuration did modify the EMG area in right SOL. This suggests that the decrease in MEP by GVS could be explained simply by a change in motoneuronal excitability. However, this does not exclude the possibility that changes could have occurred in premotoneuronal networks as well.

Importantly, direct comparison of the mean level of background EMG and the MEP amplitude might not be fair since the sizes are different from each other. Indeed, comparison between different response sizes (involving different types of motoneurons) could result in different conditioning effects, such as described by others [50, 60, 61]. We therefore also assessed the changes in H-reflex by GVS alone and observed that the GVS does not modulate the H-reflex at either delay which suggest that the modulation does not occur at motoneuronal level. Furthermore, as modulation of the H-reflex without changes in the EMG would have suggested that presynaptic inhibition in IA afferent might have been involved [47], the results gathered here suggest that the MEP modulation observed is not likely to occur through presynaptic inhibition.

Others have observed modulation of H-reflex by GVS at latencies similar to those tested here [35, 62–65] using a large number of stimuli during prone, sitting or standing positions. In the current protocol, only 10 stimuli were applied to facilitate or inhibit corticospinal pathways. This small number of repetitions may have prevented the H-reflex modulation to be observed in the current study.

### Polarity-specific responses

Previous studies had shown influence of the vestibular system on amplitude of MEP induced in the arm in a polarity non-specific manner [14]. In the current study, there was a difference in the vestibular modulation observed between cathode right/anode left and anode right/cathode left configurations. Responses observed were thus dependent on the laterality of the cathode. This finding underlies the behavioral responses induced in healthy subjects following the stimulation. Laterality of vestibular functions has previously been described in the literature (see Brandt & Dieterich [66]). Indeed, these authors reported that the vestibular sense was located in the right hemisphere of right-handers and left hemisphere of left-handers, and both contralateral and ipsilateral pathways could connect vestibular nuclei and cortex. Although it was suggested that this vestibular dominance concerned mainly ‘higher vestibular functions’ such as orientation and spatial memory, rather than vestibular reflexes, the difference in MEP modulation observed in response to the side of the cathode being to the right or to the left could also reflect this dominance.

### Possible sites of interaction

To assess whether vestibular interaction could occur at the level of the motor cortex, we measured the GVS-evoked changes in the short-latency and long-latency cortical facilitation of the H-reflex in the right SOL. Short-latency facilitation by TMS is known to reflect the excitability of the fastest conducting, corticomotoneuronal cells, and a change occurring in this pathway is likely to reflect changes of excitability occurring at motor cortical level [55, 56, 58]. This is further supported by the observation by Nielsen et al. [67] that the earliest H-reflex facilitation induced by TMS begins ~1–2 ms later than the facilitation induced by transcranial electrical stimulation that produces D-waves, which corroborates other studies showing that TMS over the leg motor cortex predominantly induces I-waves rather than D-wave (s) [67–69]. Short-latency facilitation is also known to be difficult to observe in the standing position. Notably, Petersen et al. [58] reported that, whereas the short-latency facilitation could be evoked in almost all participants during walking and dynamic plantar flexion, it was only observed in 10/17 participants in tonic plantarflexion and in 8/14 participants during quiet standing. In the current study, the short-latency facilitation could be observed in 8/10 participants and in these GVS could not change the amplitude of the short-latency facilitation of the SOL H-reflex by TMS. Thus, GVS could not change excitability of corticomotoneuronal cells at the motor cortex level.

We also tested the long-latency facilitation of the H-reflex, believed to be mediated by indirect descending pathways acting at premotoneuronal sites [55]. Long-latency H-reflex facilitation is easier to observe during standing and facilitation was observed in all participants. Cathode right/anode left GVS could decrease the facilitation of the SOL H-reflex at ISI = 40 ms (Fig 5B). This confirms that, at this delay, the vestibular output modulated the MEP at premotoneuronal level. However, although data does not demonstrate involvement at cortical level, the limitation of the methodology does not exclude this possibility. Indeed, we only used one of the cortical measures available in electrophysiology. The use of other measures such as EMG suppression by subthreshold TMS [70–73] effects of coil orientation [74–76], short-interval intracortical inhibition and short-interval intracortical facilitation [77, 78] would shed more light on the actual mechanisms occurring at cortical levels. In addition, experiment with transcranial electrical stimulation [67] could also be used to support involvement of subcortical mechanisms for the MEP modulation at 40 ms.

Furthermore, we assessed the corticospinal influence originating from M1 only and the role of other cortical areas cannot be ignored. Notably, the somatosensory and PIVC receive important vestibular input and their activity have been shown to be modulated by GVS [79, 80]. These cortical areas also project to motor cortex and through intracortical and intercortical pathways these areas could modulate the corticospinal neurons. This in turn may influence the amplitude of MEP or of the H-reflex facilitation.

### Variability of the responses

Variability was observed between individuals in the GVS-induced EMG responses and MEP-modulated responses which could be partly explained by the head facing forward in our study. Indeed, previous studies had also underlined the variability of responses in SOL and TA with such head position [33], which might be related to the activation of different muscles to achieve a sway in the same direction. In the current study, the subject had a frontal head orientation which leads binaural GVS to induce mainly medial-lateral responses. Such configuration is not optimal to observe clear EMG responses to GVS in SOL and a posture with the head turned could enhance EMG responses to GVS in SOL [59]. This posture could also enhance responses on MEP. However, it was decided to use this set-up so we could extend our findings to functional tasks such as walking (usually performed with frontal head orientation). Another limit was the fixed stimulus intensity (3.5 mA) as this feature might not be optimal for each subject. This choice was based on our previous data [40] showing that 3.5–4 mA was enough to induce a vestibular response in all subjects and ensured the activation of the vestibular system without being too uncomfortable.

### Influence of asymmetrical stance on the results

Another point to consider is that our subjects were asked to lean slightly to the right and forward. Although it is not likely to have biased EMG responses to GVS, as an asymmetrical stance doesn’t affect the direction of the responses to GVS, or the sway towards the anode [81], the weight shift may have influenced the muscle responses. Notably, the same group showed that when applying a 3s- or a 1s-GVS of 1 mA while the participant adopts an asymmetrical stance, the loaded leg produced greater lateral force and the magnitude of vertical force response was greater than during symmetrical standing. In our study, even though the EMG level was controlled, the load on each foot was not, so the influence of this position on our vestibular responses remains unknown. Body orientation is another confounding factor. Zhang et al. [82] and Mullick et al. [83] reported that the vestibular output is involved in the control of body orientation with respect to the direction of gravity. In the current study, subjects leaned slightly to the right to increase the tonic activity of the right SOL, but also changing slightly the body orientation. This asymmetry may have influenced the observed responses. Hence, although asymmetrical stance enabled easier induction of MEP it might have tampered the vestibular output. The effects observed may thus be quite task-specific and not be readily generalized to other standing tasks.

### Neuronal substrate underlying the premotoneuronal and motoneuronal effect

Studies from Kennedy and collaborators (2004a), assessing the effects of GVS bipolar and binaural stimulation on motor units showed significant modifications in the onset of activation and in the initial firing frequency of gastrocnemius muscles, suggesting a change in the gain of the presynaptic inhibitory mechanisms that act on the motor pool [84]. Moreover, assessment by the same group of the effect of monaural monopolar as well as bipolar binaural GVS on H-reflex amplitude further suggest involvement of presynaptic inhibition in conveying the effect of galvanic vestibular stimulation onto the motor pool [85]. As it has been previously shown that the corticospinal tract could also modulate presynaptic inhibition, this latter mechanism might be a common mechanism through which vestibular stimulation influences corticospinal output at premotoneuronal level. Although, there is evidence in the literature to support that vestibulospinal projections can also modulate presynaptic inhibition [34] these studies do not show that vestibulospinal tract can affect presynaptic terminals of the cortical fibers. Furthermore, in the current study, the lack of change in H-reflex amplitude in the presence or absence of EMG modulation does not support the involvement of presynaptic inhibition in GVS- modulation of MEP.

Another likely premotoneuronal target might be the Ia inhibitory interneurons, enabling reciprocal inhibition. Indeed, previous studies showing that both corticospinal and vestibulospinal tracts make connections with Ia inhibitory interneurons in cats [86, 19] and in humans [34, 87]. However, there is no evidence that both corticospinal and vestibulospinal mechanisms act synergistically on the Ia inhibitory interneurons. In order to be taken into consideration, the influence of both cortical and vestibular systems should be assessed further.

### Relevance of the findings

Our data add to studies previously performed where modulation by vestibular input of corticospinal output was assessed. Results from the current study suggest that modulation of MEP in SOL muscles by GVS is likely occurring at pre-motoneuronal as well as motoneuronal levels, which echoes previous studies. Indeed, Suzuki et al. [14] demonstrated that galvanic vestibular stimulation (GVS) could facilitate motor evoked potentials (MEPs) in arm muscles through the cervical interneuronal system [14]. Furthermore, Guzman-Lopez et al. [15] showed that caloric vestibular stimulation could modulate MEP amplitude in neck muscles. Results from the latter study suggested that this vestibular modulation of corticospinal SCM control likely occurred at cortical levels.

Furthermore, the findings of the present study are potentially relevant to understand postural reactions during frontal-plane perturbation. Indeed, recent studies have shown that in response to lateral perturbations during gait, the ongoing stance is shortened [88, 89]. In our study, the perturbation mimics a forward plane perturbation. We may then speculate that the inputs arising from the vestibular afferents could then contribute to a postural reaction that could limit the impact of the perturbation, by decreasing corticospinal activation of the SOL through pre-motoneuronal or motoneuronal pathways and produce a decrease in SOL EMG response on one side. Thus, the vestibular modulation in SOL MEP could contribute to appropriate balance reaction following frontal plane perturbation during standing.

This research might have important clinical implications. Indeed, studies using animal models have shown that after vestibular loss there was an increased amplitude of both reactive and anticipatory postural responses. Such hypermetric postural responses lead to postural and gait deficits [5, 6, 90]. Similarly in patients with bilateral or unilateral peripheral vestibular loss, postural reactions are not adapted to the direction of the perturbation [91] and the amplitude of the response is exaggerated [7], even though the latency of the responses remains stable [92]. The current study has identified specific modulation, mainly inhibitory, of the corticospinal output by increased or decreased vestibular inputs. These interactions could be involved in maintaining postural control during voluntary movement, following frontal perturbation and could also be key to understand dysfunction after vestibular loss. However, further investigation in subjects with vestibular deficits are needed to conclude on the role of these interactions in balance deficits.

## Conclusion

The aim of this study was to identify the nature and timing of vestibulocortical interactions underlying balance control during standing. Our findings showed that vestibular output suppresses corticospinal activity on the side of the cathode both at premotoneuronal (ISI = 40ms) and motoneuronal levels (ISI = 130ms). With our experimental paradigm no interaction could be evidenced at the level of the motor cortex but highlighted possible premotoneuronal site of interaction. This constitutes a step in unraveling the specific pathways and convergence patterns of vestibular and corticospinal signals involved in balance control in healthy young adults. The identification of the interaction between these pathways could lead to a better understanding of motor deficits after vestibular dysfunction.
